# Rhinitis, sinusitis and ocular disease – 2088. Evaluation of blood markers for determination of atopy in Indian patients with symptoms of respiratory allergy

**DOI:** 10.1186/1939-4551-6-S1-P167

**Published:** 2013-04-23

**Authors:** Vattikonda Sathavahana Chowdary

**Affiliations:** 1 Ent, Head & Neck Surgery, Allergy Clinic, Apollo Hospitals, Jubilee Hills, Hyderabad, India

## Background

The cause of allergy-like symptoms may be due to IgE mediated allergy, but also because of other mechanisms.There is a need to find a simple and convenient test to identify/exclude atopy for a correct and proper patient management in patients with respiratory allergies in India.

## Methods

105 patients (18-60 years) with a clinical diagnosis of asthma and/or rhinitis, based only on clinical history and physical examination,visiting an Otolaryngology department in a tertiary hospital were included in the study. Phadiatop, a test with extensive data published in the West showing >90% sensitivity and specificity, was used as the gold standard for atopy. Phadiatop measures IgE antibodies to a balanced mixture of common environmental allergens relevant for the geographical area and the age of the patients (adults). For each patient the result of Phadiatop was compared with: I. Clinical history (Hx), II. Total IgE level (T-IgE) and III. Absolute Eosinophil Count (AEC).

## Results

Phadiatop revealed atopy in 69% (72/105) of the patients, which means that approx. 1/3 of the patients diagnosed with IgE-mediated allergy by Hx in fact were not allergic.The sensitivity for atopic disease was 90.3% and 33.3% for T-IgE and AEC, respectively. Among the 33 patients with positive Hx but negative Phadiatop; 7 patients had increased T-IgE level and 8 had increased AEC, however only one patient with both tests positive. The efficiency of T-IgE and AEC to diagnose atopic allergy was 83.8% and 46.7% respectively, compared with Phadiatop.

## Conclusions

Diagnosing patients with allergy-like respiratory symptoms is difficult without using any tests; by Hx only, the allergy diagnosis was overestimated in more than 30% of the cases, which is in agreement with earlier findings. Although valuable, a good clinical history is not definitive. The results from this study suggest that Phadiatop could be a very useful and cost saving initial test in India when differentiating between atopic and non-atopic allergy, but further investigations of the Indian allergen coverage may be needed.

